# Angiosarcoma of the Face: A Case Study and Literature Review of Local and Metastatic Angiosarcoma

**DOI:** 10.1155/2021/8823585

**Published:** 2021-07-01

**Authors:** Hugo Lara-Martinez, Molly Weinberg, Praneeth Baratam, Jeffrey Horn, Kristine Ward, Michael Styler

**Affiliations:** ^1^Dartmouth Hitchcock Medical Center, 1 Medical Center Drive, Lebanon NH, USA; ^2^Drexel Hematology/Oncology, 230 N Broad St, Philadelphia PA, USA

## Abstract

Angiosarcomas are vascular malignancies with a tendency to spread extensively both locally and systemically. We report a case of cutaneous angiosarcoma of the face in a 53-year-old man that was originally misdiagnosed as an abscess. Initially small, the lesion enlarged over a four-to-six-month period and began to bleed. Two shave biopsies were performed that returned a diagnosis of angiosarcoma. The patient underwent radical resection and lymph node dissection, which revealed positive margins and ten of forty-six positive lymph nodes. The patient was treated with paclitaxel and concurrent radiation therapy (RT). Restaging scans showed a new sclerotic lesion of the T10 vertebra, three hepatic lesions, and an adrenal lesion, all concerning for metastasis. Biopsy of one of the hepatic lesions was consistent with metastatic angiosarcoma. In this review, we discuss the presentation of cutaneous angiosarcoma, the importance of early diagnosis, and the treatment options available for metastatic disease that has failed first-line chemotherapy.

## 1. Introduction

Angiosarcomas are rare vascular malignancies that present with metastases in approximately 20% of patients more often than with other types of soft tissue sarcoma [1]. Spread to local lymph nodes occurs more often with angiosarcoma than with other types of soft tissue sarcomas. Resection is the initial therapy for local disease, and chemotherapy such as paclitaxel and doxorubicin is commonly used for recurrent or metastatic disease. Tyrosine kinase inhibitors, angiogenesis inhibitors, and combinations of the aforementioned with cytotoxic agents are currently being used in clinical trials [2]. The case presented here demonstrates the importance of early, accurate diagnosis of angiosarcomas due to their tendency to metastasize widely. Following treatment failure, determination of second-line therapy in the recurrent or metastatic setting is difficult at present due to the lack of prospective studies.

## 2. Case Presentation

A 53-year-old African American male with a past medical history of chronic obstructive pulmonary disease (COPD), current smoker (20 pack-years), was initially presented in July 2016 to the dermatology department with a fungating mass on the left nasal sidewall. The mass appeared four to six months prior to presentation as a small lesion. At that time, multiple physicians told him that it was an abscess; however, it was never drained. The lesion then continued to enlarge and started bleeding significantly. The bleeding became so severe that he began having symptoms of shortness of breath with exertion, fatigue, and weakness that required an intensive care unit (ICU) stay for symptomatic anemia.

**Figure 1:**
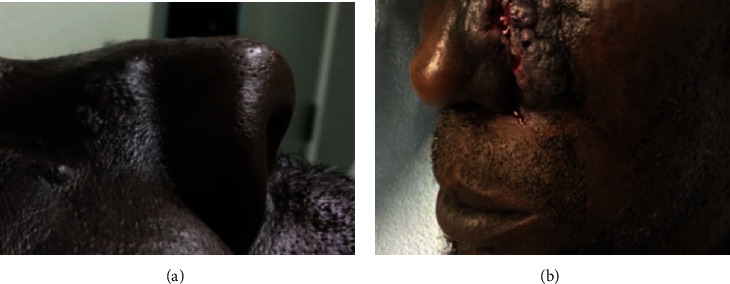
(a) Right nasal sidewall of patient's angiosarcoma. (b) Left nasal sidewall of patient's angiosarcoma.

On exam, there were several hyperpigmented patches with papules extending to the right nasal sidewall and a 4 mm flesh-colored papule on the right cheek (Figure 1(a)). There is a 10 cm fungating verrucous plaque with central ulceration and friable areas on the left nasal sidewall extending to the left cheek (Figure 1(b)).

**Figure 2:**
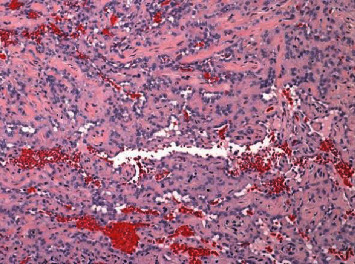
H&E staining of patient's left facial angiosarcoma.

**Figure 3:**
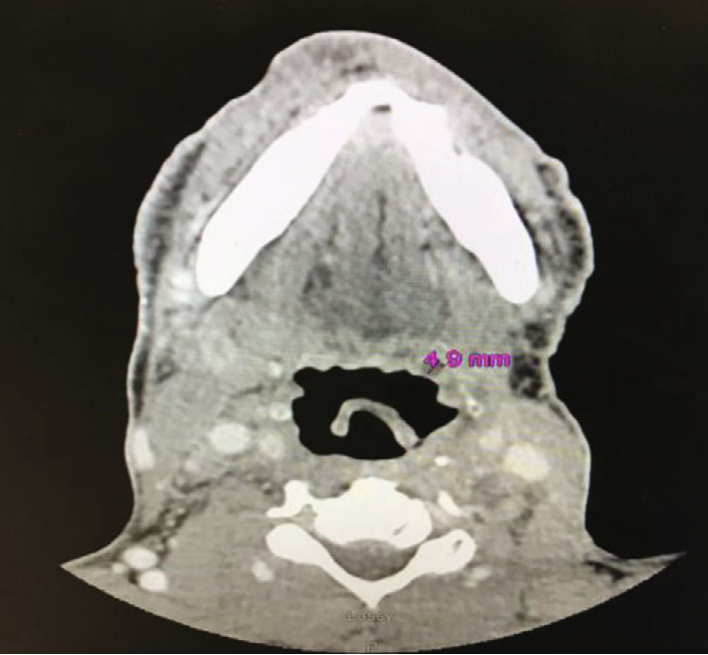
CT neck with contrast imaging of left nasal mass.

**Figure 4:**
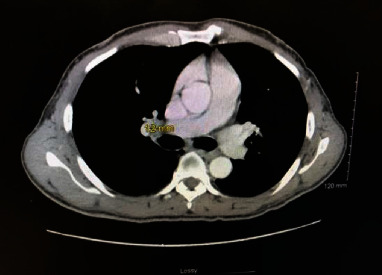
CT chest with contrast imaging of right hilar mass.

The patient had significant left submental and postauricular lymphadenopathy. Dermatology performed two shave biopsies of the mass (left nasal sidewall and left nasal bridge) and cauterized the bleeding areas. Results returned as angiosarcoma as seen in (Figure 2). Punch biopsies were then performed by dermatology to evaluate for multifocality, and the patient was referred to oncology for further management. He was staged with a CT maxillofacial, CT neck, CT chest, CT abdomen and pelvis, and MRI brain (Figure 3). Radiation oncology and surgery teams evaluated the patient. Staging workup was completed, and tumor was staged at III (T1b, N1M0). Incidentally, a right hilar lung mass and node were found (Figure 4). A core biopsy of this lesion showed lung adenocarcinoma, staged at Ia (T1b, N0, M0); no additional mutation studies were ordered at that time.

In September 2016, the patient underwent a left radical maxillectomy, left excision of facial tumor, left orbital exploration with medial canthotomy and medial canthus repair, resection of left lacrimal apparatus, endoscopic left anterior and posterior ethmoidectomy, and left modified neck dissection. He subsequently underwent skin grafting to the left face from an anterolateral thigh flap graft. Management of the lung adenocarcinoma was deferred until recovery from facial surgery. Pathology results from the resection showed positive margins and gross residual tumor. Ten of the 46 lymph nodes dissected were positive.

Following the surgery, hematology and oncology along with radiation oncology planned for chemoradiation with paclitaxel. There was, however, a gap in care for a few months due to social issues with the patient. He also required dental extractions prior to starting radiation. In February 2017, the patient was able to have two dental extractions and was cleared to start radiation treatment. He began treatment with CT simulation in March 2017 and completed 8 cycles of paclitaxel 50 mg/m^2^ and 66.6 Gy of RT in two phases over 37 fractions in May 2017. He experienced mucosal toxicity and skin desquamation from the radiation treatment, but this completely recovered over three months. Definitive management of the lung adenocarcinoma was deferred due to not being able to tolerate both treatments simultaneously.

Following completion of chemoradiation, restaging PET CT in September 2017 showed small residual FDG activity with a SUV of 5.6 in the left maxilla and avid known RUL mass abutting the medial pleura now larger in size. His lung adenocarcinoma was upstaged to IIIA (T2, N2, M0). He received CT simulation of the lung lesion in October 2017 followed by 9 cycles of carboplatin/paclitaxel with RT and two additional doses of carboplatin/paclitaxel consolidation.

**Figure 5:**
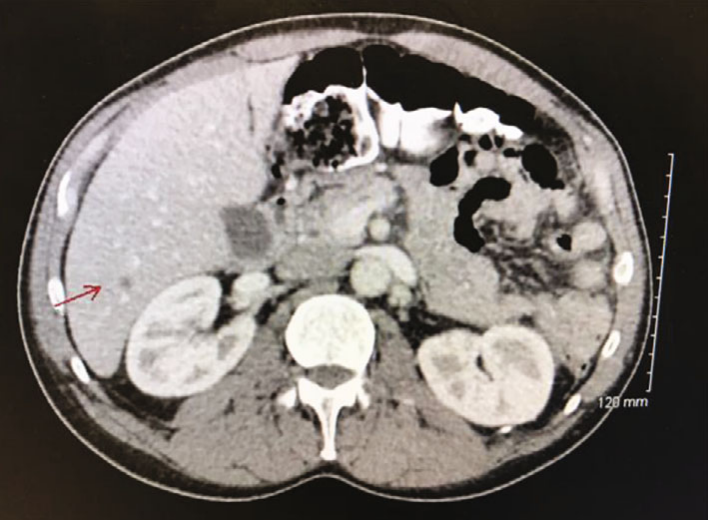
CT with contrast imaging of liver metastasis (arrow).

In April 2018, restaging scans showed an interval decrease of the right hilar mass but a new nonspecific T10 sclerotic lesion, three new hepatic lesions, and a new left adrenal gland lesion, all concerning for metastasis (Figure 5). CT of the maxillofacial region also showed progression of soft tissue in the right maxillary sinus with slightly increased overlying infiltration of the right premalar and right paranasal soft tissues concerning for malignancy and effacement of the left piriform sinus which is new.

A hepatic lesion was biopsied in June 2018 that was consistent with metastatic angiosarcoma. The patient was then lost to follow-up.

## 3. Discussion

Angiosarcomas are a very aggressive and rare type of soft tissue sarcoma accounting for 1.6% of soft tissue sarcomas [3]. Location of the primary tumor can vary; however, they most commonly occur in the head and neck, followed by the breasts and extremities [4]. They appear to have a male predilection with most studies demonstrating a 2 : 1 distribution [5]. They can appear at any age, but the cutaneous form seems to be more common in older white males [4]. The exact aetiology is unknown, and most arise spontaneously.

There are some risk factors associated with developing angiosarcomas which include chronic lymphedema, radiation, exogenous toxins such as vinyl chloride, arsenic, and thorium, neurofibromatosis type 1, Maffucci syndrome, and mutations on BRCA 1 or BRCA 2 [6].

The diagnosis of angiosarcomas can often be delayed due to misdiagnosis as a fungal infection, bacterial infection, Kaposi's sarcoma, melanoma, or ulcers since they have a wide range of appearance [4]. Clinical presentation can vary depending on time of presentation.

Angiosarcomas can initially appear as bruises or raised purplish papules but can further grow and look like ulcerated, fungating masses [7]. They often haemorrhage as with our patient.

The pathology of angiosarcomas can differ but often is characterized by irregular, anastomosing, and dilated vasculature similar to that in benign lymphangioendothelioma [8]. They can express the endothelial markers CD31, CD34, vWF, vascular endothelial growth factor (VEGF), and U europaeus agglutinin 1 [9]. These can aid in the diagnosis.

Angiosarcomas have a poor prognosis compared to other soft tissue sarcomas, with a five-year survival of 35% [10]. Survival is higher in those with localized disease. Some factors associated with a worse prognosis include old age, high tumor grade, large tumors greater than five centimeters, metastatic disease, and poor performance status [6, 11, 12].

Interestingly, it should be noted that ever-smoking is a poor prognosis and associated with a decrease in disease-specific survival in patients with angiosarcomas compared to nonsmokers [13]. This could be related to the fact that smoking has been linked to progression and metastasis likely due to its effect in the cell cycle and apoptosis [14]. It is unclear if smoking cessation can improve outcomes, but it has been shown that smoking cessation for 3 months can improve pulmonary function capacity [15].

Treatment varies depending if disease is localized or metastatic. The treatment of choice in patients with localized disease is radical surgery with complete resection [16]. It is recommended to resect with wide margins due to the aggressiveness of the disease. Patients with limited disease should receive adjuvant radiation therapy at 50 Gy with wide treatment fields due to the high likelihood of local recurrence [16]. A retrospective analysis of treatment of angiosarcoma of the scalp and face showed that combination of surgery and radiation offered the best survival at 45.8% at 2 years, whereas either treatment alone only afforded 11.1% survival at 2 years [17]. In patients with later stage or advanced disease, the use of chemotherapy using cytotoxic agents is appropriate [4].

In patients with metastatic disease, cytotoxic chemotherapy is the primary treatment option. First-line treatment is a single-agent regimen with paclitaxel, doxorubicin, bevacizumab, sunitinib, or sorafenib [16].

A retrospective study of the EORTC soft tissue and bone sarcoma group found paclitaxel to be an active agent in angiosarcomas particularly in those of the face and scalp with a response rate of 75% and time to progression of 9.5 months [18]. However, Italiano et al. [19] have shown that using single-agent doxorubicin or weekly paclitaxel appears to have similar efficacy in metastatic angiosarcomas, but cutaneous angiosarcomas respond more favorably to weekly paclitaxel. Many patients, however, have treatment failure or recurrent disease. One medication that has shown to be promising is pazopanib, a multiple target tyrosine kinase inhibitor that has received approval to treat certain soft tissue sarcoma. In a phase III randomized trial comparing pazopanib vs. placebo in patients with metastatic angiosarcoma, it was shown a progression-free survival of 4.6 months in the pazopanib group compared to the placebo 1.6 months [20]. Another small trial that was conducted by Pasquier et al. demonstrated a strong synergism between propranolol and vincristine in an in vitro model and interestingly lead to 100% response in patients with inoperable angiosarcoma [21].

Our patient has unfortunately failed therapy with paclitaxel-based regimens used as an adjunct to radiation or along with carboplatin as part of his lung cancer treatment, although the metastatic recurrence happened after a 3-month long hiatus with no therapy due to patient compliance issues. He now has distant metastasis to multiple sites. We are considering the use of pazopanib or restarting weekly paclitaxel therapy if he can tolerate another course of treatment. We are also considering radiation therapy if he chooses a palliative option. Due to the lack of prospective studies and the limited number of cases, it can be difficult to determine the standard of care for treatment of angiosarcomas.

Targeted therapies have shown promising results, such as the use of VEGF inhibitors such as bevacizumab, PDGF inhibitors such as olaratumab, and tyrosine kinase inhibitors such as pazopanib [20].

## 4. Conclusion

A case of angiosarcoma of the face has been presented in this report. This case helps us understand the clinical presentation of angiosarcomas and the importance of arriving at the correct diagnosis initially. It is also important to be diligent with nonhealing ulcers or wounds. In early stages, localized radical resection and radiation therapy may be a sufficient form of treatment. In metastatic disease, however, doxorubicin or paclitaxel are suitable as first-line agents. One should note that paclitaxel has a better response in the cutaneous form of angiosarcomas, especially of the scalp and face. Novel targeted therapy looks promising for the future of treatment.

Since our patient has failed first-line paclitaxel-based therapy and is now presenting with diffuse metastatic disease, a trial of pazopanib appears to be the appropriate next action.

## Data Availability

The data used to support the findings of this study have not been made available because of hospital closure.

## References

[1] Morrison W. H., Byers R. M., Garden A. S., Evans H. L., Ang K. K., Peters L. J. (1995). Cutaneous angiosarcoma of the head and neck. A therapeutic dilemma.

[2] Malangani M. A., Rosen M. J. (2012). Sabiston textbook of surgery.The biological basis of modern surgical practice. *Sabiston Textbook of Surgery.The Biological Basis of Modern Surgical Practice*.

[3] Rouhani P., Fletcher C. D. M., Devesa S. S., Toro J. R. (2008). Cutaneous soft tissue sarcoma incidence patterns in the U.S.: an analysis of 12,114 cases.

[4] Young R. J., Brown N. J., Reed M. W., Hughes D., Woll P. J. (2010). Angiosarcoma. *The Lancet Oncology*.

[5] Wang L., Lao I. W., Yu L., Wang J. (2017). Clinicopathological features and prognostic factors in angiosarcoma: a retrospective analysis of 200 patients from a single Chinese medical institute.

[6] Fayette J., Martin E., Piperno-Neumann S. (2007). Angiosarcomas, a heterogeneous group of sarcomas with specific behavior depending on primary site: a retrospective study of 161 cases.

[7] Dossett L., Harrington M., Cruse C., Gonzalez R. (2015). Cutaneous angiosarcoma. *Current Problems in Cancer*.

[8] Shustef E., Kazlouskaya V., Prieto V. G., Ivan D., Aung P. P. (2017). Cutaneous angiosarcoma: a current update. *Journal of Clinical Pathology*.

[9] Ohsawa M., Naka N., Tomita Y., Kawamori D., Kanno H., Aozasa K. (1995). Use of immunohistochemical procedures in diagnosing angiosarcoma. Evaluation of 98 cases.

[10] Fury M. G., Antonescu C. R., Van Zee K., Brennan M. E., Maki R. G. (2005). A 14-year retrospective review of angiosarcoma: clinical characteristics, prognostic factors, and treatment outcomes with surgery and chemotherapy.

[11] Park J. T., Roh J. L., Kim S. O. (2015). Prognostic factors and oncological outcomes of 122 head and neck soft tissue sarcoma patients treated at a single institution. *Annals of Surgical Oncology*.

[12] Lee B. L., Chen C. F., Chen P. C. H. (2017). Investigation of prognostic features in primary cutaneous and soft tissue angiosarcoma after surgical resection. *Annals of Plastic Surgery*.

[13] Yeang M. S., Tay K., Ong W. S. (2013). Outcomes and prognostic factors of post-irradiation and de novo sarcomas of the head and neck: a histologically matched case-control study. *Annals of Surgical Oncology*.

[14] Pezzuto A., Citarella F., Croghan I., Tonini G. (2019). The effects of cigarette smoking extracts on cell cycle and tumor spread: novel evidence. *Futur. Sci. OA*.

[15] Pezzuto A., Carico E. (2020). Effectiveness of smoking cessation in smokers with COPD and nocturnal oxygen desaturation: functional analysis. *The Clinical Respiratory Journal*.

[16] National Comprehensive Cancer Network (NCCN) (2018). NCCN Clinical Practice Guidelines in Oncology. *Soft tissue sarcoma (Version2.2018)*.

[17] Ogawa K., Takahashi K., Asato Y. (2012). Treatment and prognosis of angiosarcoma of the scalp and face: a retrospective analysis of 48 patients. *British Journal of Radiology*.

[18] Schlemmer M., Reichardt P., Verweij J. (2008). Paclitaxel in patients with advanced angiosarcomas of soft tissue: a retrospective study of the EORTC soft tissue and bone sarcoma group. *European Journal of Cancer*.

[19] Italiano A., Cioffi A., Penel N. (2012). Comparison of doxorubicin and weekly paclitaxel efficacy in metastatic angiosarcomas. *Cancer*.

[20] van der Graaf W. T. A., Blay J. Y., Chawla S. P. (2012). Pazopanib for metastatic soft-tissue sarcoma (PALETTE): a randomised, double- blind, placebo-controlled phase 3 trial.

[21] Pasquier E., André N., Street J. (2016). Effective management of advanced angiosarcoma by the synergistic combination of propranolol and vinblastine-based metronomic chemotherapy: a bench to bedside study.

